# MCA-NMF: Multimodal Concept Acquisition with Non-Negative Matrix Factorization

**DOI:** 10.1371/journal.pone.0140732

**Published:** 2015-10-21

**Authors:** Olivier Mangin, David Filliat, Louis ten Bosch, Pierre-Yves Oudeyer

**Affiliations:** 1 Flowers Team, Inria, Bordeaux, France; 2 U2IS, ENSTA ParisTech, Université Paris Saclay, Saclay, France; 3 Centre for Language and Speech Technology, Radboud University, Nijmegen, Netherlands; University of Sheffield, UNITED KINGDOM

## Abstract

In this paper we introduce MCA-NMF, a computational model of the acquisition of multimodal concepts by an agent grounded in its environment. More precisely our model finds patterns in multimodal sensor input that characterize associations across modalities (speech utterances, images and motion). We propose this computational model as an answer to the question of how some class of concepts can be learnt. In addition, the model provides a way of defining such a class of plausibly learnable concepts. We detail why the multimodal nature of perception is essential to reduce the ambiguity of learnt concepts as well as to communicate about them through speech. We then present a set of experiments that demonstrate the learning of such concepts from real non-symbolic data consisting of speech sounds, images, and motions. Finally we consider structure in perceptual signals and demonstrate that a detailed knowledge of this structure, named *compositional understanding* can emerge from, instead of being a prerequisite of, global understanding. An open-source implementation of the MCA-NMF learner as well as scripts and associated experimental data to reproduce the experiments are publicly available.

## Introduction

Whether they are considered to ground the meaning of words or to give structure to our high level perception, concepts are an essential aspect of human cognition as well as a desirable feature for robots and other artificial cognitive systems. Yet the exact definition of what concepts are, let alone, the question of how they may be acquired by an agent in interaction with its physical environment are still mostly unanswered.

### Multimodal concepts

Concepts may be defined as *mental representations*. What these mental representations are, would they be forged by a human or a robot, is however not firmly defined. Thus, before building models of the acquisition of concepts, and therefore indirectly of the concepts themselves, some aspects must be clarified. In the paper we propose a perspective where learning concepts is viewed as discovering patterns in a flow of multimodal perception, a view that is in line with the embodied cognition principle (see [1]) and perceptual concepts as the ones from Barsalou [2] (for a more detailed review see [3]).

More precisely we consider the notion of *multimodal concepts* for several reasons. First, many concepts do not lie in a single perceptual modality. For example, the emergence of the concept of *dog* is not only related to the ability to recognize pictures of dogs in the visual modality, but also to the sound of a dog barking and the touch of a dog’s fur. Furthermore, many concepts cannot be completely characterized without grounding them on several modalities: the concept *metallic* cannot be characterized without taking into account its perceptual expression on several modalities (for example visual aspect, sound, touch, or taste), together with the recognition of the spoken or written word.

Another reason to consider multimodal concepts is that concepts often occur in the context of language and hence involve a linguistic modality. This aspect is emphasized in particular in the symbol grounding problem, as introduced by Harnad [4] and discussed by Glenberg and Kaschak [5]. It points out that learning language is not only about learning the signs of communication such as words, but also requires to relate them to their semantic content, that emerges from and is grounded in the interaction with the world. This process is denoted as the *semiotic association*. From that perspective, learning a concept may involve learning and relating its semantic content to a given symbol. Natural communication channels however do not contain such thing as absolute symbols but rather manifestations of these symbols: the same word is never heard the same twice and the image of that word written is not perceived the same depending on the font it is written with, the angle it is viewed from, as well as the ambient luminosity. Thus perceiving symbols is by itself an analogous problem to perceiving their meaning. Hence the symbol grounding problem becomes a weak instance of the larger problem of learning meanings in one modality and linguistic symbols in another modality. In other words the notion of symbols as invariant printed names of object is challenged and replaced by the broader notion of abstract categories. Also, human communication is not generally reduced to one modality such as speaking or writing; instead full featured communication makes extensive use of facial expressions, physical contact, and eye gaze. A famous evidence of the multimodal nature of communication was given by McGurk and MacDonald [6] and is referred as the *McGurk effect*: observing lips pronouncing *‘ga’* while hearing *‘ba’* is most often reported as perceiving the sound *‘da’* (see also [7]). In that perspective the multimodal character of natural language makes it very similar to the kind of concepts it may refer to; so that linguistic elements may be seen as multimodal concepts themselves. For these reasons we propose to study the learning of multimodal concepts that span several modalities, including linguistic and non-linguistic channels. In particular this approach emphasizes the co-organization of language and meaning, which is in line with growing evidences of the influence of language in learning concepts (see [8, 9]).

However, trying to define what it means to learn multimodal concepts reveals that this task is prone to many ambiguities. As pointed out by Belpaeme and Morse [10]: *“The challenge which cross-situational learning needs to solve is not only one of mapping a word to a meaning, but of distinguishing that meaning from possible distractors.”* Indeed, Quine’s *indeterminacy of reference* (see [11]) states that relating words to meanings when learning a foreign language is intrinsically ambiguous. On the other hand, many models of learning semantic components from one modality also encounter similar ambiguity issues. For exampe, Cederborg and Oudeyer [12] draw a parallel between Quine’s inderterminacy and ambiguity in imitation learning, that they call *the motor gavagai problem*. Another example is encountered with concepts that corresponds to categories. Indeed infants learning categories face the alternative possibilities of *thematic* and *taxonomic* associations of concepts as explained in Markman [13]: thematic association refers to the association of concepts that are related because they interact together, as *milk* and *cow*; taxonomic association refers to concepts that belongs to the same class, such as *cow* and *pig*. Other analogies can be drawn between this phenomenon and the ambiguity of word segmentation (see [14]), but also with multistability phenomenon in perception as described by Blake, Leopold, Schwarts et al. [15–17], and the *cocktail party effect* (see [18]). Many multimodal problems feature ambiguity in one or several modalities, but, somehow paradoxically, integrating information from several modalities may be efficiently used to overcome such ambiguity. In other words, considering the problem of concept learning separately in each modality suffers from the presence of ambiguities, but looking at the same problem in several modalities at the same time might help resolving that ambiguity instead of increasing it. For example the role of multimodal perceptions relatively to multistability is discussed by Schwartz et al. [17]. Similarly Schwartz et al. [19] explore the role of vision of the lips for improving intelligibility of spoken sound and Sodoyer et al. [20] present an algorithms for source separation taking advantage of audio-visual information. Finally, Massera et al. [21] demonstrate, in an other experiment, that a robot can reach better performance on a motor task when a linguistic guidance is present, even if it does not initially know the meaning of the symbols composing the guidance.

Another import benefit from taking into account the multimodal nature of perception is to increase robustness to missing data. Indeed, with multimodal data, one might infer missing information in one modality from the other modalities, which is an important property compared to unimodal data streams.

### Learning associations

A central issue toward defining the problem of learning multimodal concepts is to formalize what it means to *learn* a concept. Importantly *concepts* do not necessarily refer to an explicit representation; this notion rather targets emerging behaviors that are interpreted as the mastering of that concept. For example a child is said to master the concept *dog* not by looking into his brain for a neuron spiking each time a dog is seen but rather by its ability to relate the sight of a dog with the sound of a barking dog or the sound of the name *‘dog’*. One way of modelling this is to specify a behavioral evaluation of the learning process. In particular this paper focuses on the ability to classify stimuli in a specific way. The behavioral evaluation it uses is very similar to the one used in language games research: it consists in giving as input to the learner a cue on one modality (for example a speech utterance), and to let it behaviorally select what it refers to within a context which contains several possible references (as an image in the example).

From a machine learning perspective, unlike supervised learning, unsupervised learning, or reinforcement learning, multimodal learning is not associated to a specific class of algorithms. Indeed, multimodal data can be treated as unimodal data on which an unsupervised learning algorithm is applied (some examples provided in this paper fall under this category). It can also be considered a supervised regression problem that consists in predicting the signal in one modality while the information in the other modalities is given. The focus of this work is on learning that occurs in an unsupervised manner, that is how multimodal perception self-organizes in a way that can explain the emergence of concepts. The kind of behaviors under consideration are classification behaviors. However they do not correspond to supervised classification in machine learning, that is the association of a symbol to a given stimuli, but rather to unsupervised association between stimuli from a same semantic class. Indeed classification capabilities in children emerge from association capabilities that start from fuzzy many-to-many mappings, in the way a child groups a car with a truck instead of with a cow (as discussed by [13]).

This paper actually only focuses on one type of concepts, characterized by cross-modal associations, and a model of their acquisition. An example is the concept *dog* with its visual, acoustic, acoustic as language, and tactile manifestations. Although this notion of concept may seem very limited, we argue that the model has a large potential for generalization to other modelling domains.

In practice, we build experiments that involve two phases: an unsupervised learning phase where the system observes raw data streams, and a behavioral evaluation in which it solves the behavioral task of recognizing associations across modalities. This separation opens a perspective on the relation between the properties of the perceptual signal available during learning and the nature of the learnt concepts that is specified by the evaluation task. In particular it raises the question of the drives and cues that enable the self-organization of multimodal perception. In the case of language learning, experiments on children performed by Akhtar and Montague [22], and Smith and Yu [23] demonstrate that *cross-situational learning*, which focuses on elements that are persistent in the environment across different uses of a word, might be used by children to learn the meaning of words. Most of this work relies on cross-situational learning to explain or model the acquisition of lexicons of concepts. Other mechanisms such as *the whole object assumption*, *mutual exclusivity* (see [13]), and *conceptual reasoning* (see [24]) are also known to play a role in the process of associating linguistic labels to concepts, but are out of the scope of this paper.

### Structure and complexity

Natural perception generally consists in *complex* visual or acoustic scenes rather than in isolated pictures of objects or isolated sounds of words. In other words, an essential aspect of these scenes is their structure; and an essential capability for a learner is to leverage that structure to overcome the complexity of its perception. Such structure can however take several forms: for example a visual scene may contain several objects at various positions; a spoken sentence is composed of a sequence of words, the words themselves are made from basic phonemes and the sequence obey to a specific grammar.

A common intuition is that the complexity of the learnt concepts gradually increases along learning. In particular *cumulative learning* consists in gradually acquiring a lexicon of elements of increasing complexities, such that new elements can be obtained as the combination of simpler ones (see sec. 3–4 from [25]). Actually the definition of what *complex* and *simple* mean may by itself be ambiguous; therefore we focus in this paper on a specific structure that is the combination of several basic elements in complex scenes such as objects in an image or words in a sentence.

Motor synergies and motion primitives are other examples of building blocks that can be combined to model complex perception and action; they were introduced by both motor control theorists and roboticists (see [26]). For example Mussa-Ivaldi and Bizzi [27] interpret a group of experiments on the control system of frogs and rats as giving strong evidence that the brain uses a set of primitive force fields that are combined linearly to generate a diversity of motor behaviors. Tresch and Jarc [28] provide a more detailed review on that subject.

A higher complexity of perceptual stimuli increases the ambiguity during the acquisition of concepts. We already mentioned examples of that ambiguity such as word segmentation (see [14]), but there is also ambiguity because of the competition between acoustically close words. One way to handle that ambiguity is to frame the learning on multimodal concepts, thus relating the structure in one modality with the structure in others. For example Tuci et al. [29] provides a model of multimodal learning for symbolic language and real actions. Their experiment demonstrates that learning a compositional structure shared between action and language can allow robotic agents to achieve better generalization of the acquired motor knowledge. More precisely the linguistic input received by the system shapes a model of the structure of actions and makes the system capable of achieving behaviors that were not encountered in training.

Another interesting question arises from the notion of structured concepts and their acquisition and challenges the meanings of the words *simple* and *complex*. Wrede et al. [30] contrast *compositional* understanding, that describes an agent that is aware of the local components and their combination into a global perception or action, and *teleological* understanding, that accounts for an agent that understands perception globally. More precisely the term teleological refers to a pragmatic emphasis on using the global knowledge toward a goal, even without refined understanding of its structure. The non-literal interpretation of a formulaic expression (such as *“to kick the bucket”* meaning *“to die”*) can be understood as an example of teleological understanding, while the literal interpretation is an example of compositional understanding. Indeed, the meaning of a group of words may be different from the semantic composition of the constituent words. This forms a big issue in compositional semantics theory. According to Wrede et al. [30] the developmental path of infants goes first through teleological understanding before reaching compositional understanding. This developmental path is to contrast to the one stating that compositional understanding occurs first before any usage of the knowledge (for example as suggested by [25]).

### Contribution

In this work we introduce a model named *Multimodal concept acquisition with non-negative matrix factorization* (MCA-NMF), of the learning of cross-modal concepts through the formation of structure in multimodal low-level signals (vision, speech sounds, gestural motions). We then present experiments combining the learning of dance gestures from human demonstrations, of words from full spoken sentences, and of visual objects from images.

We demonstrate the acquisition of grounded complex concepts from raw continuous signal only, without relying on symbols to train the artificial agent. Our learning experiment exploits cross-situational associations in the perceptual training data. Language learning, and in particular the learning of words, is in MCA-NMF treated as an instance of multimodal learning. This means that the linguistic data (here speech) is not handled in a specific way but rather in a symmetric manner with respect to other perceptual modalities. Finally we explore the question of the structure and (de)composition of concepts. In particular we show that it is possible for an artificial system to discover subcomponents of perception, such as words in spoken sentences, although the system is only exposed to a task that requires to associate whole sentences to corresponding scenes.

The next section presents the experimental framework as well as the datasets used in the experiments. Section *Methods* details the algorithms that forms the MCA-NMF learner, including the low-level processing of sensor data. Section *Experiments* provides the results and their analysis for the experiments on learning multimodal concepts as cross-modal associations. It also provides two additional experiments on the discovery of words inside sentences and the explicit representation of concepts by the learner. Finally we discuss our contribution and its articulation to previous work in Section *Discussion and Perspective*.

## Materials

This section presents the experimental framework that we use to explore the question of learning multimodal concepts from perception. It first details the kind of concepts we consider, then explains the experimental setup, and finally introduces the datasets we use.

### Target concepts

This paper presents a system that discovers patterns that characterize associations in multiple modalities: that are patterns in some modality that are systematically associated with other patterns in other modalities. We perform several experiments that explore how the learner manages to represent semantic relations between the modalities. In practice we consider semantic relations that may correspond to either an *essential* relation, as the one relating the barking to the image of the dog, or a *conventional* relation as the one relating the name *‘dog’* to images of dogs.

The essential relation arises from the reality of an object that has manifestations in several modalities. Their exists such a thing as a dog that has manifestations in the visual modality as images of the dog, in the touch modality as the touch of the dog’s fur or its claws, or in the acoustic modality as the sound of the dog barking. Although not all of these manifestations occur each time the dog is encountered, they are often perceived simultaneously since they corresponds to the actual presence of the dog. On the other side, the conventional relation characterises language: the word *‘dog’* is often pronounced when a dog is present and is the object of attention. This process is extensively used by parents to teach new words to children.

Importantly, both relations manifest through the joint occurrence of frequent patterns in several modalities; in other words, both manifest through cross-situational information. Hence a mechanism leveraging such cross-situational information is able to learn both these essential and conventional relations. In the following we denote by *semantic concept* the set of manifestations of such an object in perceptual modalities, either related essentially or by convention. Additionally a semantic concept may have several kinds of manifestations in a single modality. For instance a dog is associated to both the touch of its fur and claws, or to the sound of the dog barking and the word *‘dog’*. The semantic relations we consider actually include Peirce’s icon, index, and symbol (see 3.1 in [31]). In the following, the only cue in the stimuli about the semantic relations is that related elements occur simultaneous in the various modalities; this corresponds to the cross-situational information.

### Experimental framework

**Fig 1:**
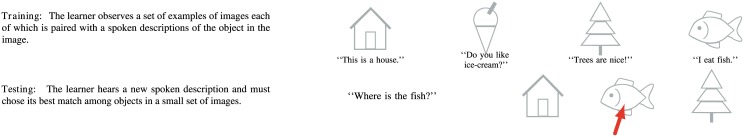
Illustration of the cross-modal classification task that models the recognition of an object from its name, after cross-modal habituation. The learner perceives multimodal signal, vision and sound in the figure, and learns the associations between the appearance of objects and the sound of their names. It then has to prove its ability to chose the right object among a small set when hearing its name. In practice we use images of objects, motion captures, and recorded spoken utterances in the experiments.

We consider the situation in which an intelligent system perceives a scene, composed for example of objects or motions, while hearing sentences that describe the scene. Such a setup is illustrated in Fig 1.

In the experiments we describe below, the modalities that the system observes can vary from one experiment to the other and it is not necessary that one modality is linguistic; however a semantic relation always exists between some elements of the different modalities. These elements might be of several natures: gestures in motions, object in visual scenes, or words in spoken utterances. We consider semantic relations as mappings between these elements: for example a word is related to a gesture, or a gesture to an object in a scene.

In the following, each experiment consists in two phases: one training phase during which the system is exposed to multimodal data and an evaluation phase in which we test the success of concept learning.

During its training the learning agent observes examples of scenes; each example is a set of one observations in each modality. In each scene only one relevant multimodal concept is present and is observed in several modalities. For example a sentence is heard containing the word *‘dog’* and a picture of a dog is seen. Although only one multimodal concept is present in each example, not all perceived elements are meaningful, that is to say related to elements in other modalities. For instance many words appear in spoken utterances that are not semantically related to anything in other modalities. For example in the sentence *“Look at the circles I do!”*, only the word *‘circle’* is related to the observed gesture. Similarly several objects may appear in the visual scene while only one is related to the subject of the sentence.

During its evaluation the learner observes a test example in only one modality. It then has to chose between several other examples in another modality, the one that best matches the test example. For example the system hears a sentence talking about a dog and has to chose between several pictures the one containing a dog. We denote this task *cross-modal classification*; it forms an evaluation protocol that does not require one modality to be symbolic.

Interestingly this experiment is very similar to the one performed by developmental psychologists to study the role of various heuristics used by children for the acquisition of words, including cross-situational information, as in the works from Markman, Yurobsky et al. [13, 32]. Unlike many computational approaches presented in previous works on multimodal learning, we present an evaluation of the performance of the learner that is not on a regular machine learning classification task. Instead the learner is evaluated on its ability to relate elements from distinct non-symbolic modalities, similarly to how one would evaluate a children. In additional experiments presented in Section *Word acquisition and recognition*, we also evaluate the emergence of the recognition of single words inside sentences. More precisely the learner is asked to recognize parts of sentences instead of full utterances and this enables to localize the perceived semantics inside the speech sentences. An experiment also studies the mapping between the internal representation of the system and the semantic information to detect the emergence of explicit representations of the learnt concepts.

### Datasets

The following experiments involve three raw modalities: motion, sound, and image.

#### Motions

**Fig 2:**
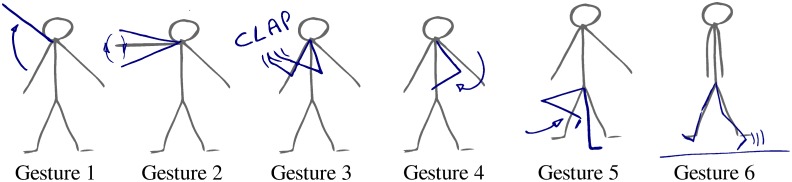
Dance motions were performed by a human dancer and perceived through skeleton tracking based on a 3D kinect sensor. The figure illustrates some of the gestures demonstrated to the learner.

In the experiments we use a dataset of choreography motions demonstrated by a human and recorded through a *kinect* device. More precisely the dataset contains a total of 1100 records, each presenting one of 10 different gestures that are spanned over one or two limbs. Fig 2 illustrates the kind of gestures that compose the datasets. Typical gestures are the *walk* gestures, involving the two legs, in which the human demonstrator mimics walking in place, or the *left hello* in which the demonstrator moves his left arm pretending to say *‘hello’*. The data is publicly available and presented in more details at Mangin [33] and is used by Mangin and Oudeyer [34, 35].

#### Speech

The acoustic records used in the following experiments are taken from the Caregiver dataset (see [36]), provided by the ACORNS project (see [37]). It is composed of 1000 utterances containing 13 keywords. Each utterance is spoken by 4 speakers in English adult directed speech. In the experiments we only use utterances from one speaker. An example of sentences used in the dataset is *“Angus is lazy today.”* where the keyword is *‘Angus’*. Other examples of transcriptions from utterances from the dataset are given in Table 1.

**Table 1:** Transcriptions from ten random examples from the Acorns Caregiver dataset from Altosaar et al. [36]. Keywords are identified in bold font.

We take a **bath**
To put it in the **bath** isn’t funny either
The **shoe** is a symbol
Now **mummy** is losing her patience
**Daddy** comes closer
**Angus** takes off her shoe
**Daddy** never calls
She sits on a **nappy**
Now everybody is in the **car**
Where is the **nappy**

#### Images

**Fig 3:**
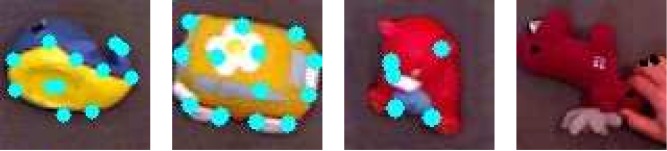
Example frames from the image dataset. These frames feature the following objects: *blue whale*, *yellow car*, *teddy bear* and *moose*. Color circles corresponds to local descriptors detected by the system. Interestingly, the hand of the operator appears in some pictures as in the last one. The area observed by the system is actually larger than the one represented in the figure.

Pictures used in the experiments were acquired as frames from an interaction with an iCub robot, through an RGBD sensor (red, green, and blue camera coupled with a depth sensor). The acquisition was performed and is described in more details by Lyubova and Filliat [38]. Each image contains one visual object and possibly the hand of the operator. In the experiments we use images from a subset of 10 objects each appearing in more than a thousand frames. During the acquisition, the objects are moved and rotated. Hence they are presented from distinct points of view and they may be partially cluttered by the hand of the operator. Examples of frames from the dataset are presented in Fig 3.

## Methods

### Open-source code to reproduce the experiments

The implementation of the MCA-NMF and the code used in the experiments presented in this paper are available publicly and openly (BSD license) on http://github.com/omangin/multimodal. It consists in a set of tools that implement the MCA-NMF system, including the NMF algorithm and code to achieve multimodal learning with it, as well as the scripts that correspond to the experiments presented here and produce their results. It includes the methods to process sound and motion data. Features extracted from the datasets are also publicly available as http://dx.doi.org/10.5281/zenodo.29600, http://dx.doi.org/10.5281/zenodo.29602, and http://dx.doi.org/10.5281/zenodo.29607. The code is meant for the reproduction of the experiment we presented as well as for the development of new experiments based on the same framework.

### The MCA-NMF model

This section presents the algorithmic tools behind MCA-NMF. They are based on the nonnegative matrix factorization algorithm presented by Paatero, Lee et al. [39, 40] and that enables for example unsuppervised learning of internal representations from textual documents (see [41]). We use it very similarly to Ten Bosch, Driesen, Mangin et al. [34, 42, 43].

**Fig 4:**
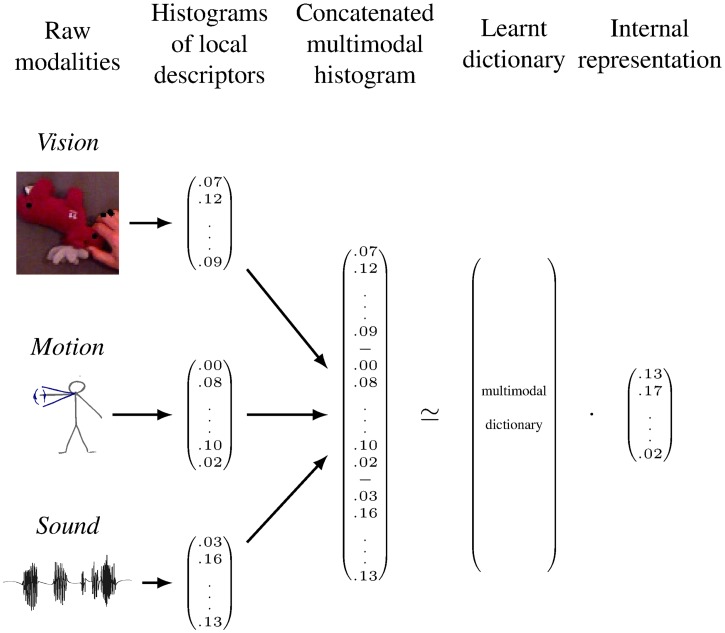
The MCA-NMF system processing of data. First raw data from each modality is transformed into a histogram of local descriptors, that is a vector of nonnegative values. Then the histograms from each modality are concatenated into a histogram representation of the multimodal perception. During its training the MCA-NMF system uses a set of perceived example, each represented by such an histogram, to learn a multimodal dictionary that captures multimodal patterns. The system then uses the dictionary to transform perception into an internal representation. The internal representation can be obtained with all modalities observed (as on the figure) or with only a subset of modalities observed as explained further.

The first part of this section presents the learning of a multimodal dictionary; it is then explained how the learned dictionary provides a representation of data that is not bound to any modality; in the following this representation is referred to as the learner’s *internal representation* of data. Finally we explain how the learner can transform data from one or several modalities to an internal representation or to an expected representation in unobserved modalities. Fig 4 provides an overview of the transformations from raw data to higher level representations computed by the learning agent.

We consider a setting in which the learner observes samples in several modalities. For example, the system visually observes objects while hearing a spoken description of the scene. The perception of the samples in each modality is represented by a vector *v*_*a*_, where *a* denotes the modality (for example the system observes the objects as *v*_*image*_ and the sound description as *v*_*sound*_). Importantly the vectors representing all samples from a modality must have the same dimension. Although this constraint is not trivial to satisfy, details about such representations for the modalities used in the experiments are given in Section *Signal representation*.

#### Learning a dictionary of multimodal components

We call *components* primitive elements that are mixed together into observations, in the same way that phonemes can be seen to combine together into a word or a sentence. Compared to the common context of clustering, this notion of component is more general: observations are mixtures of several components at the same time, instead of being just a noisy observation of one centroid.

The learner implemented by MCA-NMF builds a dictionary of multimodal components according to the following model: it searches *k* components, each represented by a vector *w* ^*j*^ (*j* from 1 to *k*), such that each observed example *v* ^*i*^ verifies:

$$\begin{array}{c} v^{i}≃\sum_{j=1}^{k}h_{j}^{i}w^{j} \end{array}$$ (1)

where $h_{j}^{i}$ are coefficients and ≃ denotes a notion of similarity between matrices that is defined below. This is equivalent to clustering when the *w* ^*j*^ are the centroids and for each *i* only one $h_{j}^{i}$ is nonzero and equals 1. We consider a more general case where *w*_*i*_ and $h_{j}^{i}$ are only constrained to be nonnegative.

In the following, the set of *n* examples is represented by a matrix *V* of shape *d* × *n* (each example is a column of *V*), the set of components by a matrix *W* of shape *d* × *k*, called *dictionary*, and the coefficients by a matrix *H* of shape *k* × *n*. The previous equation, that models the objective of our learner, can thus be re-written for all observations as:

$$\begin{array}{c} V≃W\cdot H \end{array}$$ (2)

In order to fully define the reconstruction error between *V* and *W* ⋅ *H*, we use a variant of the Kullback-Leibler divergence often called generalized Kullback-Leibler or I-divergence. The Kullback-Leibler divergence is originally an information theoretic measure of the similarity between two probability distributions. In particular this choice is relevant for the representations based on histograms of features that we use in the experiments and that are very sparse and high dimensional. The I-divergence is defined, for two matrices *A* and *B* of same shape, as *D*_*I*_(*A*‖*B*) given by Eq (3).

$$\begin{array}{cc} D_{I}(A∥B) & =\sum_{i=1}^{d}\sum_{j=1}^{n}(A_{i,j}\ln(\frac{A_{i,j}}{B_{i,j}})-A_{i,j}+B_{i,j}) \end{array}$$ (3)

Similarly to what happens with the Frobenius norm, the NMF problem is convex in each matrix (*W* and *H*) but not the joint problem on (*W*, *H*). In this paper in order to minimize *D*_*I*_(*V*∣∣*W* ⋅ *H*), we use the algorithm, based on multiplicative updates of *W* and *H*, that was originally presented in Lee and Seung’s paper [40]. This algorithm consists in alternating the two update steps from Eq (4) where ⊛ and / denote Hadamard’s (coefficient-wise) product and division on matrices. The algorithms stops after a maximum number of iteration is reached or the loss function has stabilized.

$$\begin{array}{cccc} H & \leftarrow H⊛\frac{W^{T}\frac{V}{W\cdot H}}{W^{T}\cdot 1} & \;W & \leftarrow W⊛\frac{\frac{V}{W\cdot H}H^{T}}{1\cdot H^{T}} \end{array}$$ (4)

#### NMF to learn mappings between modalities

Previous section explains how, for a given set of observations from several modalities that is represented by a matrix *V*, the NMF algorithm can learn a dictionary *W* and a coefficient *H* matrices such that training examples are well approximated by the product *W* ⋅ *H*.

We actually consider the case of data coming from several modalities (three in the example). More precisely we assume the data matrix *V* is composed of column vectors *v* such that:

$$v=(\begin{array}{c} v_{mod1}\\ v_{mod2}\\ v_{mod3} \end{array})\;\text{and}\;\text{thus}\;V=(\begin{array}{c} V_{mod1}\\ V_{mod2}\\ V_{mod3} \end{array}).$$

The minimization of the I divergence induces a trade-off between error in one modality relatively to others. In order for the error in each modality to be treated on a fair level by the algorithm it is important that the average values in the representations are of similar magnitude. It can be easily obtained by normalizing data in each modality. In the following experiment data from each modality is composed of histograms and thus normalized according to its average 1-norm.

Since the observations, that is to say the columns of *V*, are composed of several modalities, the dictionary *W* also splits into several parts each corresponding to one modality. In other words each components can be seen as the concatenation of several parts: one for each modality. For example if the data is composed of three modalities: *mod*1, *mod*2, and *mod*3, there exist matrices *W*_*mod1*_, *W*_*mod2*_, and *W*_*mod3*_ such that:

$$\begin{array}{c} W=(\begin{array}{c} W_{mod1}\\ W_{mod2}\\ W_{mod3} \end{array}). \end{array}$$

In the following we interpret the columns of the matrix *H*, as an internal representation of the data by the learner. For example, an internal representation *h* is induced by an observation in modality one such that *v*_*mod1*_ = *W*_*mod1*_ ⋅ *h*, or one in both modalities one and three by:

$$\begin{array}{c} (\begin{array}{c} v_{mod1}\\ v_{mod3} \end{array})=(\begin{array}{c} W_{mod1}\\ W_{mod3} \end{array})\cdot h. \end{array}$$

Interestingly, it is possible to use the learned dictionary to compute an internal representation of an example, even if the example is only observed in a subset of the modalities. Given an example observed only in one modality, *v*_*mod1*_, one can search for an *h* such that *v*_*mod1*_ is well approximated as *W*_*mod1*_ ⋅ *h*. More precisely this is equivalent to finding an *h* solution of:

$$\begin{array}{c} \underset{h}{\arg\min}\;D_{I}\left(v_{mod1},W_{mod1}\cdot h\right) \end{array}$$ (5)

The NMF algorithm we use in these experiments actually alternates steps minimizing *D*_*I*_(*V*∣∣*W* ⋅ *H*) with respect to *W* and *H*. Solving Eq (5) is equivalent to the NMF problem with respect to *H* only; therefore, it can be obtained with the same algorithm, but only using the steps that update *H*. In theory this approach scales to any number of modalities although the experiments presented here only test it on numbers from two to four.

**Fig 5:**
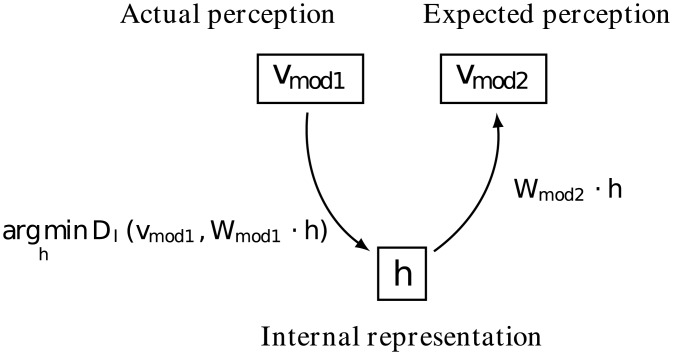
Once the system has learnt the dictionary (*W*_*mod1*_ and *W*_*mod2*_), given an observation *v*_*mod1*_ in one modality it can reconstruct the corresponding internal representation as well as the expected perception in another modality.

Finally it is also possible to reconstruct a representation of the data that the system would expect in a modality, given observations in other modalities. For that, from an observation featuring a subset of the modalities, the system fits an internal representation *h* using the method described previously. Then it can reconstruct the expected representation in an unobserved modality (for example the third modality, *mod3*) by computing the product *W*_*mod3*_ ⋅ *h*. This forms a framework, illustrated in Fig 5, that uses a learned multimodal dictionary to transform data from modalities to internal representations or expected data in other modalities. It enables a large set of experiments as illustrated in the following.

#### Cross-modal classification without symbols

**Fig 6:**
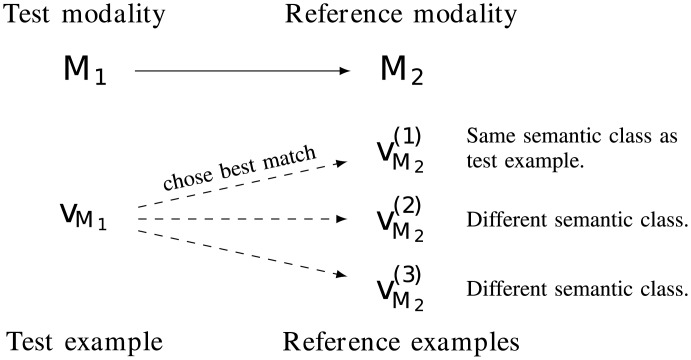
The learner is tested on its ability to relate an observation of a test example in one modality to the right reference example in another modality.

The system is trained on various combinations of either two or three modalities. The modalities might be denoted as *Motion* or *M*, *Sound* or *S*, and *Image* or *I*. After being exposed to a set of training multimodal examples, the system is tested as follows: it observes a new example, called *test example* in a subset of its modalities and has to chose the best match among several examples observed in other modalities, which are denoted as *reference examples*. An illustration of that process is given by Fig 1. For example, the system is trained on sound and image and tested by hearing a sentence (the test example) and having to chose among a set of images (the reference examples) the one that is best described by the heard sentence. Another possibility is to train the system on motions, sounds, and images, and test its ability to chose from several sentences the one that best describes a pair of a motion and an image that it observes. We denote such settings by the notation: *M*1 → *M*2, where *M*1 represents the modality or modalities in which the test example is observed, called *test modalities*, and *M*2 the modality or modalities, denoted as *reference modalities*, in which a best matching example must be chosen among a set of reference examples. For example hearing a sentence and choosing the best matching object from images is denoted by Sound → Image or S → I. Viewing an object and a gesture and finding the best matching sentence amongst examples is denoted by M, I → S. The testing process is illustrated in Fig 6.

Section *The MCA-NMF model* explains how to use NMF on multimodal data, to learn a dictionary and the associated *internal representation* and finally how to transform data either from one modality to another, or from a modality to the internal representation (see also Fig 5). We use that mechanism as a basis to implement a classification behavior for the learner. More precisely this refers to the *‘chose best match’* operation illustrated in Fig 6. In particular the issue is that the data to compare, that is representations of test and reference examples, are of different natures because they come from distinct modalities. We use the ability of the learner to convert perception to its internal representation as well as to other modalities to achieve the comparison behavior.

**Fig 7:**
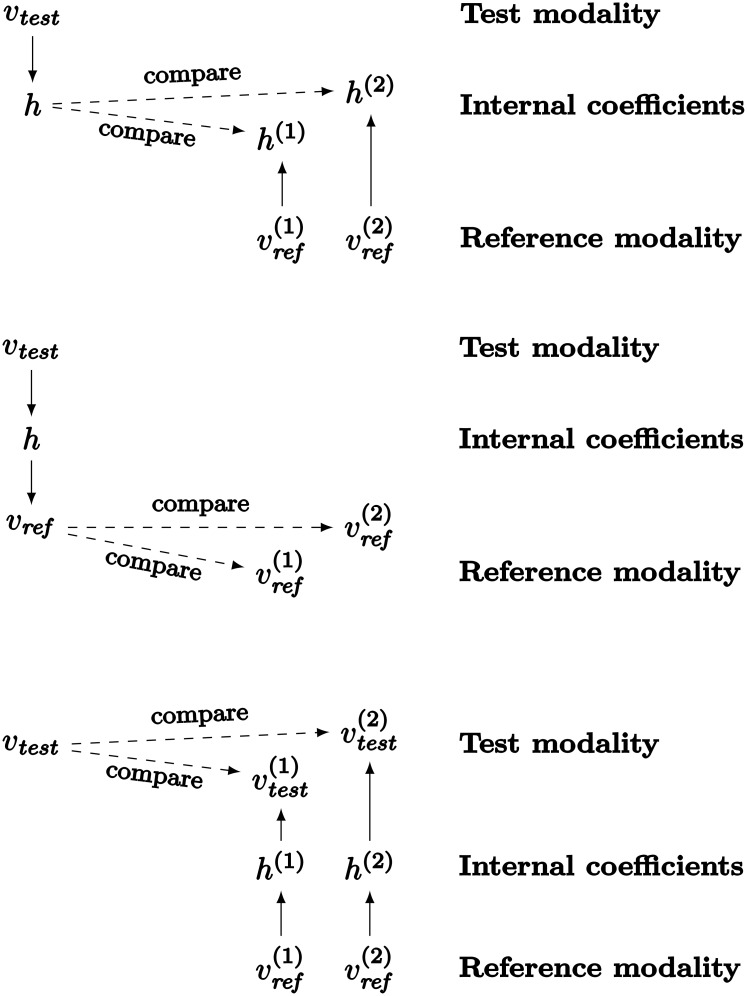
The multimodal classification task may be implemented as three different comparisons by MCA-NMF. On top, internal coefficients computed from the test example are compared to those computed from each reference example. In the middle, an expected representation of the test example in the reference modality is computed. Then this representation is compared to the actual perceptions of reference examples. Finally at the bottom, expected representations of the reference examples in the test modality are computed and compared to the actual perception of the test example.

To perform the comparison the MCA-NMF system can either:

- compute an internal representation of the test example, compute internal representations of the reference examples, and then compare these internal representations. Top of Fig 7 illustrates this process.
- compute an internal representation of the test example, use it to generate an expected representation in the reference modality, and compare it to the reference examples. Middle of Fig 7 illustrates this process.
- compute internal representations of reference examples, for each of them compute an expected representation in the test modality, and compare them to the test example. Bottom of Fig 7 illustrates this process.

The choice of one of these methods is referred as *the modality of comparison*. This is however not sufficient to fully define the system: in order to be able to chose a best matching reference example, the system needs a metric to perform the comparison. Several metrics exist. The choice and the resulting efficiency of one metric are however highly dependent on the modality of comparison, as shown by the following results. We consider the following common metrics.

- **Euclidean distance**
- **Kullback-Leibler or I-divergence** The Kullback-Leibler and I-divergences are introduced by Eq (3). In the following we denote its usage as I-divergence (I-div.). By default the divergence from the test example to a reference example is computed; however since it is not symmetrical, we also experimented with the reversed divergence (that is to say the divergence from a reference example to the test example) and a symmetrized divergence obtained as: $D_{sym}\left(x∥y\right)=\frac{1}{2}[D(x∥y)+D(y∥x)]$. None of the three approaches was systematically better in our experimentation.
- **Cosine similarity** The cosine similarity is not a metric but can be used to compare vectors; it ranges from −1 to 1 and the biggest the value is, the most similar the vectors are. It is defined for two vectors *x* and *y* ∈ ℝ^*d*^, ⋅ denoting the scalar product, as:
  $$\begin{array}{c} \text{cosine}\_\text{similarity}\left(x,y\right)=\frac{x\cdot y}{∥x∥∥y∥} \end{array}$$

Other choices are possible. In our experiments, many modalities are represented by histograms, or concatenation of histograms, that are of high dimension. Unfortunately the Euclidean norm looses its intuitive meaning in such spaces of very high dimension and with vectors that represent histograms, hence the I-divergence and the cosine similarity.

In the following, MCA-NMF is evaluated on its recognition success rate. It is defined as the proportion of correct recognition by the system: a recognition is correct when the system choses a reference example matching the semantic concept from the test example.

### Signal representation

Each of the three raw modalities used in the experiments is represented in a specific way. However all the representations are similar in their approach, all data is thus represented as nonnegative vectors, and share the important property of being additive that next paragraph describes. The nonnegative and additive properties are required by the NMF algorithms and are thus limitations to the scope of its applications.

#### Additive property

Importantly, the following representations have a common additive property, that directly comes from the use of histograms of local events. For example in the acoustic modality, if two words, which representations are *w*_1_ and *w*_2_, are concatenated into an utterance, which representation is denoted as *s*, then

$$s≃\lambda w_{1}+\left(1-\lambda \right)w_{2}$$

where 0 < λ < 1. The approximation actually ignores events such as coarticulatory effects across word boundaries. This important property transforms the sequencing operation into a convex combination. It therefore transforms a sentence into a mixture of its words, and similarly a word into a mixture of phonemes. Similarly the juxtaposition of several parts of an object is represented as the convex combination of the representations of each part. Several gestures combined in a motion are also approximately represented as the convex combination of the representations of the gestures.

#### Motion

In order to represent the recorded motion as vectors of nonnegative values in a way that makes it possible to use the algorithm presented in previous section we introduced the *histograms of motion velocity* representation in Mangin and Oudeyer [34]. In particular this representation consists in a simple histogram based representation of motion that can be seen as a rough approximation of the phase diagram of the dynamics of one body joint.

**Fig 8:**
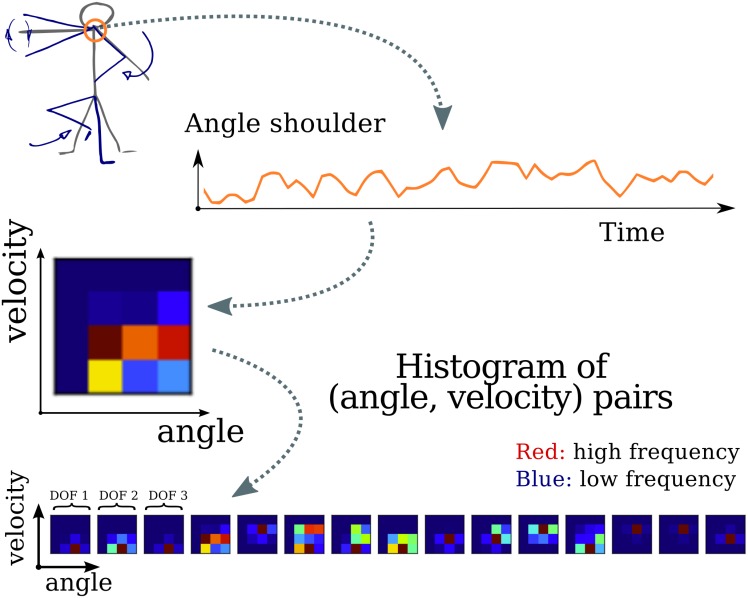
Human motions are abstracted as concatenated histograms on joint positions and velocities. In the final histograms, frequencies are represented through colors, x and y axis correspond respectively to values of angles and velocities. (Best seen in color.)

A kinect device captures the motions of a human demonstrator as trajectories in angle and angle velocity spaces of several articulations of the human body. Each trajectory on a specific body articulation (or degree of freedom) is considered separately and the entire sequence of angles and velocities is transformed into a histogram, represented by a fixed length non-negative vector. Vectors obtained for each degree of freedom are then concatenated into a larger vector as illustrates Fig 8. The device only captures angles and delayed velocities are computed to achieve better robustness to noise in the angle sequences. More precisely ${\dot{x}}_{t}=x_{t}-x_{t-d}$ is used to compute the velocities, instead of being restrained to the case where *d* = 1.

In Mangin and Oudeyer [34], we explore and compare various alternative approaches to transform angles and velocities sequences into histograms. In the following experiments we use angle-velocity histograms, that is to say histograms built on the joint space of angle and velocity. Furthermore we build histograms over a vector quantization that is an adaptive binning process using a k-means algorithm.

Representing motion data by separate histograms on each degree of freedom leads to two approximations: 1. for a given measurement in the trajectory, information about dependency between different degrees of freedom is dropped; 2. the sequential information between measures for a given degree of freedom is dropped. These simplifications are actually similar to successful ones from other fields, some of which we use for the sound and visual modalities, as detailed further. Indeed Ten Bosch et al. [42] have demonstrated that, even if sequential information may appear necessary in language, and especially in speech utterances, very good word discovery can be achieved without considering all this sequential information. Both in text classification and in computer vision *bag-of-words* techniques also achieve good performances by dropping positional information of extracted local features (see [44, 45]). Finally using histograms built on joint angle positions and velocities is similar to representing transitions in angle space. By representing the sequence through its transition we approximate it by a Markovian process. Such an approximation is quite common in the gesture recognition and motion planning literature (see [46, 47]). In the experiments, this process leads to a representation of motions that is of dimensionality 450.

#### Sound

In the following experiments we use the same representation of sound as in the works of Ten Bosch, Van Hamme, Driesen et al. [42, 48, 49]. Histograms of acoustic co-occurrences (HAC) were introduced as a representation of sound that is based on acoustic events. Similarly to the motion representation we have presented, it discards most of the sequential information of the acoustic events; it however considers co-occurrences of pairs of acoustic events and uses a static approach to codebook construction.

**Fig 9:**
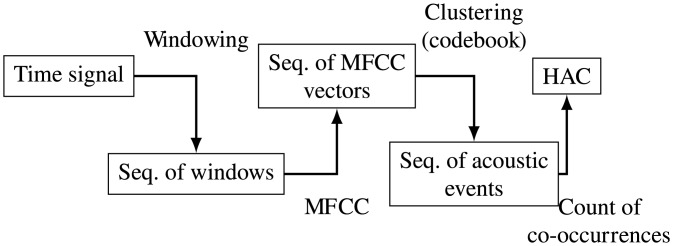
Sequence of transformations from raw (time sequence) acoustic signal to histograms of acoustic co-occurrence (HAC) representation.

The outline of the transformation from raw sound to HAC representation is given in Fig 9. The main steps are explained in more details below. The perception mechanism first segments the acoustic signal into a sequence of short time windows; it then computes Mel-frequency cepstral coefficients (MFCC) for each window. This transforms the original signal into a sequence of MFCC vectors. Additionally we consider dynamic information on top of the sequence of MFCC vectors of dimension 22; discrete derivatives are computed as defined by Driesen [50] and denoted by the Δ operator to form the ΔMFCC and ΔΔMFCC. This transformation is analogous to the delayed velocities introduced previously to represent motions.

Then, the system computes three codebooks with a k-means algorithm on a dataset of spoken language, for basic MFCC vectors and their Δ and ΔΔ transformations, as described in Driesen [50]. Following the implementation of Ten Bosch et al. [42], the sizes of the codebooks are *k* = 150 for MFCC vectors and Δ and *k* = 100 for the ΔΔ vectors. The codebooks are used to convert the three sequences of MFCC vectors and their Δ and ΔΔ transformations into a sequence of acoustic events: each cluster, that is to say each element of a codebook defines an acoustic event; each time window is thus transformed into three discrete events corresponding to the clusters in which fall the three vectors associated to that time window.

The last step consists in removing most of the temporal information by building histograms of event occurrences and co-occurrences, with the exception that the latter are expected to cover the within-phone dynamics. This process happens on top of the three sequences of discrete acoustic events obtained from vector quantization. More precisely, co-occurrence histograms are simply histograms of the successive occurrences of pairs of events. This procedure is done twice: once with a frame lag (event lag) of 2 (20 ms), and the second time with a frame lag (event lag) of 5 (50 ms). In total this gives a HAC vector of 2 + 2 ⋅ (150^2^ + 150^2^ + 100^2^) = 110002 entries.

What is denoted as HAC representation in the following is actually the concatenation of co-occurrence histograms for each one of the events categories, that is to say MFCC, ΔMFCC, and ΔΔMFCC events. The final dimensionality of the representation is 110,002; more detail on the HAC representation is given by Driesen, Van Hamme, Mangin et al. [48, 50, 51].

#### Image and video

The visual frame are transformed with the same tools as in Lyubova and Filliat [38]. More precisely, two types of *local features* are extracted from the pictures: SURF descriptors (see [52]) and HSV (hue, saturation, value) of superpixels.

As described by Bay et al. [52], SURF features are descriptors computed around points of interest in an image. They enable to transform an entire image into a set of small descriptors that are invariant to change in scale and rotation of the image. To obtain the HSV descriptors, areas with relatively homogeneous colors are grouped into what is called *superpixels* (see [53]); then each area is represented as the triplet of its hue, saturation and value.

Both methods lead to the representation of an image as a set of local descriptors. Then the descriptors of both types are quantized by incrementally learning growing dictionaries of features. This algorithm used is a variant of k-means, very similar to the one presented in Filliat [54] and for sound in Mangin et al. [55]. This process is the analogous to the grouping of MFCC vectors into acoustic events described in previous section.

Finally the occurrences of local events are counted and summarized in a histogram, that is a nonnegative vector of dimensionality around 50,000.

## Experiments

We consider semantic associations between elements of the acoustic, visual, and motion modalities; more precisely we define a semantics as an artificial mapping between acoustic words, visual objects, and gestures. An example of such a mapping is given in Table 2. Each triplet of word, gesture, object forms a semantic concept. The data used to train the MCA-NMF model is composed of sentences, motions, and images; each sentence contains one of the keywords, each motion features one gesture, each image an object. Finally the gesture, the word, and the object from an example belong to the same semantic concept, which implements the cross-situational manifestation of the semantics.

**Table 2:** We form arbitrary multimodal concepts by associating an object with a keyword and a gesture. The table presents a list of such associations. The limbs on which the motions occur are also mentioned. The system then observes the keywords as spoken sentences containing the keyword, objects as images, and gestures as human demonstrations perceived by a motion capture system.

Keyword	Object	Motion	
		Limb(s)	Description
shoe	blue octopus	both legs	squat
nappy	teddy bear		walk
book	pink octopus	right leg	raise heel toward left knee
daddy	yellow car	both arms	clap
mummy	blue whale		mimic paddling left
Angus	blue-eyes-green-yellow	right arm	mimic punching
bath	orange fish		starts horizontal and goes from side to front
bottle	squirrel	left arm	starts horizontal, forearm goes down to 90°
telephone	mouse		waving arm
car	cube		hello sign

In the testing phase of the following experiments the system is presented ten potential referrent, each of a different class among the ten possible semantic classes. Thus a random choice would lead to a rough 10% success rate. When not specified otherwise, we use a default value of *k* = 50 as the number of elements in the dictionary learnt by the NMF algorithm with 50 iterations, although a number of 10 is generally already close to convergence.

### Motion and spoken utterances

The first experiment (also detailed in [35]) compares the various evaluation methods, that is the alternative modalities of comparison and metrics. In this experiment the learner is trained on two modalities: demonstrated motions and spoken utterances. It also validates the capabilities of the MCA-NMF learner to acquire multimodal concepts that appear in a cross-situational way in training data. Table 3 presents classification success rates for various combinations of comparison modalities and metrics. These results demonstrate that the system is capable of learning aspects of the semantic associations. If the system is trained on a dataset where no semantic association exist between the two modalities (such a dataset can be obtained by choosing a random motion and a random utterance for each demonstration), it generally scores around 0.11 (Note that this is not 0.1 because the distribution of sound examples from the Caregiver dataset is not exactly uniform.). Additionally the result shows for example that the sound modality, mainly because of its very high dimension, is not a good choice for the comparison, specially when the comparison is performed with the Euclidean metric. Indeed the choice of the metric to use is highly dependent on the nature of the data in the modality; therefore, using the internal representation is a way to only use one metric. Therefore the following experiments focus on performing the comparison on the internal representations, using cosine similarity as a metric. The main interest of proceeding that way is that the comparison is the same, regardless of what the test and reference modalities are.

**Table 3:** Success rates of recognition of the right reference example from a test example. The values are given for many choices of the reference test and comparison modalities and various measures of similarity. The results are obtained by averaging on a ten fold cross-validation; baseline random is in that case 0.11.

Modality			Success rate		
Test	Reference	Comparison	I-div.	Euclidean	Cosine
Sound	Motion	Internal	0.608	0.612	0.646
		Motion	0.552	0.379	0.444
		Sound	0.238	0.126	0.208
Motion	Sound	Internal	0.610	0.704	0.830
		Sound	0.106	0.090	0.186
		Motion	0.676	0.642	0.749

Table 4 presents analogous results in the case were symbolic labels are included in the training data. Indeed one could expect that adding symbols to the training data would increase its structure and thus ease the learning of the concepts in comparison to purely non-symbolic data. The labels are included similarly to the experiments from Mangin, Driesen et al. [34, 56]: the label of each example is transformed to a vector of 10 binary values with zeros everywhere except for a one at the index corresponding to the label. This binary vector is concatenated to the vector representing the example. Thus this setup is equivalent to adding a third modality, which contains disambiguating symbols, in order to improve the learning. The results from Table 4 illustrate the fact that the system does not clearly take advantage of this additional information. An interpretation of these results is that the system is already capable of dealing with the ambiguity and is not helped by such additional symbolic information. However the relevance of such comments is limited to the current algorithm and implementation of MCA-NMF.

**Table 4:** There is no systematic improvement of the recognition rate when unambiguous symbols are added to the training data. The table represents the same success rates as previously (see Table 3) but with a learner that observed symbolic labels representing the semantic classes during training. The results are obtained by averaging on a ten fold cross-validation; baseline random is in that case 0.11.

Modality			Success rate		
Test	Reference	Comparison	I-div.	Euclidean	Cosine
Sound	Motion	Internal	0.387	0.699	0.721
		Motion	0.543	0.261	0.424
		Sound	0.136	0.089	0.131
Motion	Sound	Internal	0.573	0.620	0.702
		Sound	0.114	0.090	0.122
		Motion	0.519	0.469	0.552

### Sound, motion, and image

The experiments in this section compare the performance of learners trained on various combinations of two or three modalities among sound, motion, and image. In particular it explores the impact of adding modalities during training that do not occur during testing. In the following we only use the comparison on internal representation. The main interest of proceeding that way is that the comparison is the same, regardless of what the test and reference modalities are. Results are presented together with box plots corresponding to 20 repetitions of the experiment with random label associations, test set, train set, and reference examples.

More precisely several setup are presented, including learning from motion and sound, as well as from image and sound, as previously, but also learning from motion and image, and finally learning from the three modalities at the same time. For each of these choices of learning modalities, several setup are possible for the test phase, specially when the three modalities are present during training: these include testing on the recognition of one modality from another (for example Image → Sound) but also from two modalities to another (for example Image,Motion → Sound), or conversely one modality to two (as in Motion → Sound,Image).

**Fig 10:**
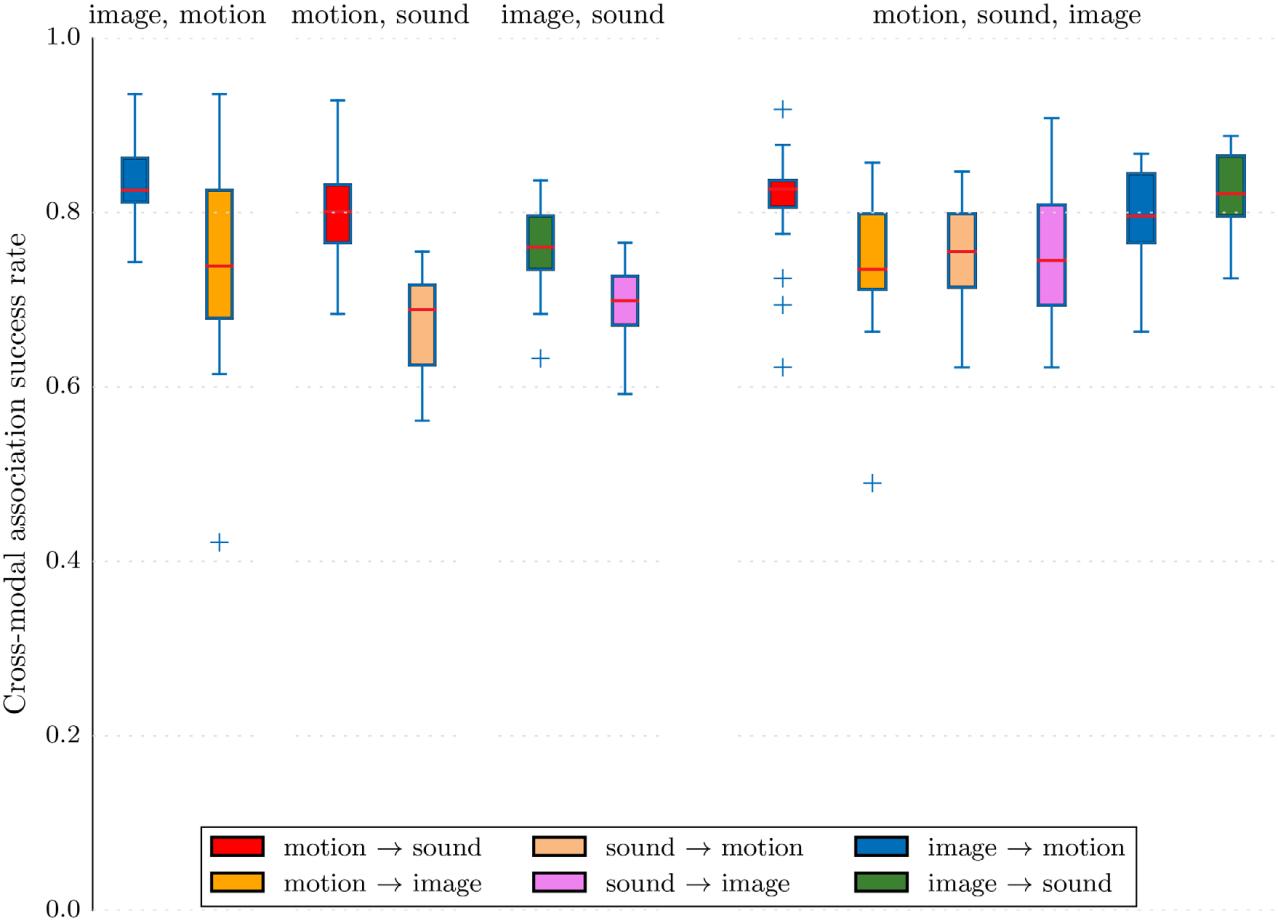
An additional modality during training slightly improves results in average. The box plot represents classification success rates for various experiments where two or three modalities are used for training. Each plot corresponds to the use of a subset of modalities during training: the first three plots use two modalities and the last one use three modalities. Each plot contains boxes representing the average success as well as quantiles and extreme values through cross-validation for various testing setups of the form *A* → *B*. There are only two testing setups when only two modalities are used for training, and six when three modalities are used for training.

**Fig 11:**
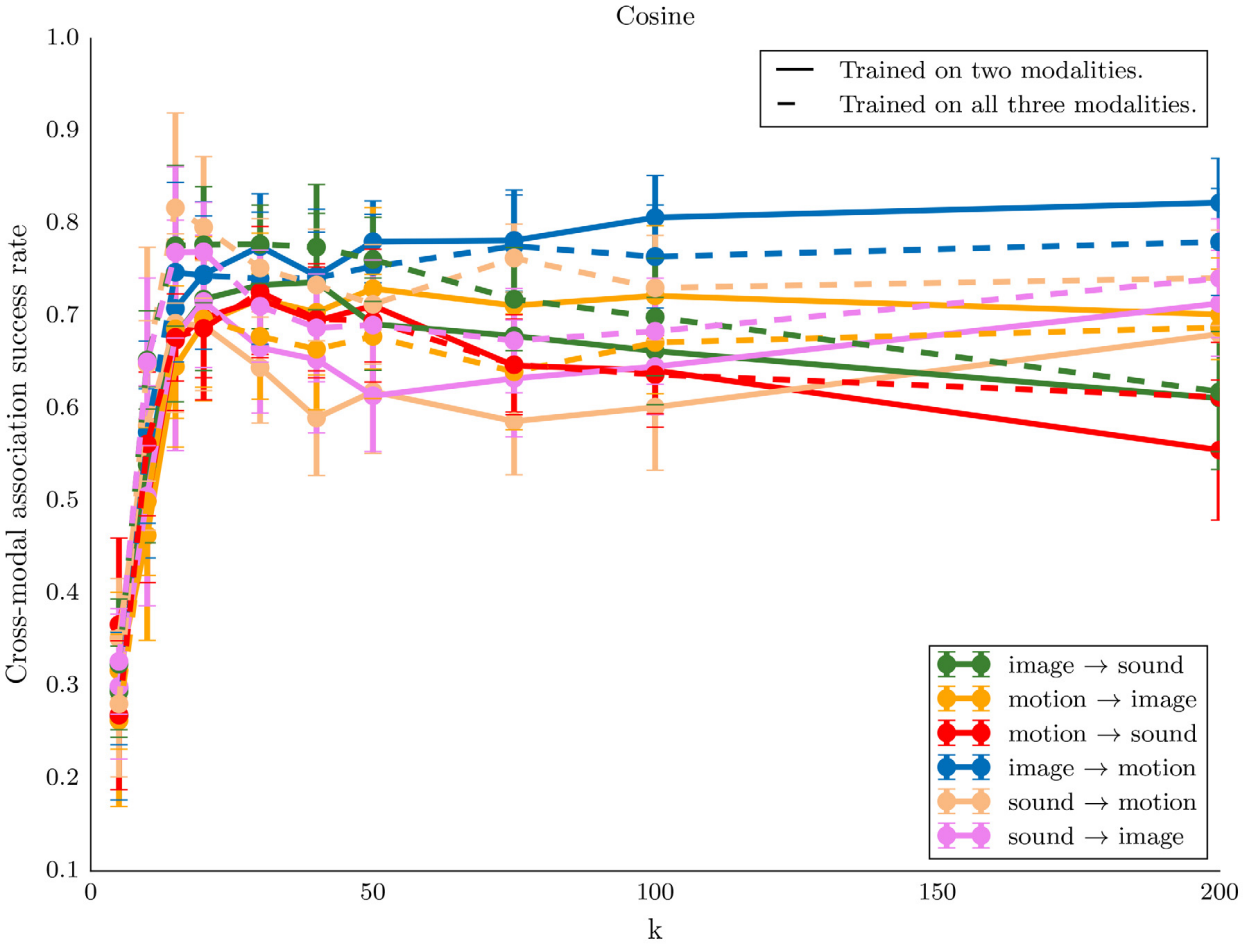
With both two (full lines) and three (dashed) modalities during training, the classification success rates are similar and good for high enough value of the number *k* of elements in the NMF dictionary. The plots demonstrate that the success rate is quite stable above a minimum value of *k*.

Fig 10 various setup of type *A* → *B*, both for the case where *A* and *B* are the only modalities present in the training and the case where a third modality is also present during training. The results demonstrate that MCA-NMF is capable of learning the semantic concepts even when more than two modalities are present. There is no major difference between the cases of two and three modalities: the system neither benefits noticeably from the third modality nor does it suffer from the increased dimensionality of the data. However, since we fix the number *k* of elements in the NMF dictionary, the results could come from the fact that when the system is trained on three modalities, the dimension of the dictionary becomes insufficient to encode non-meaningful aspects of the three modalities. Therefore Fig 11 present the same experiment for various values of *k* in order to interpret more precisely the previous result. The comparison confirms the fact that the system mainly behaves similarly with two or three modalities.

**Fig 12:**
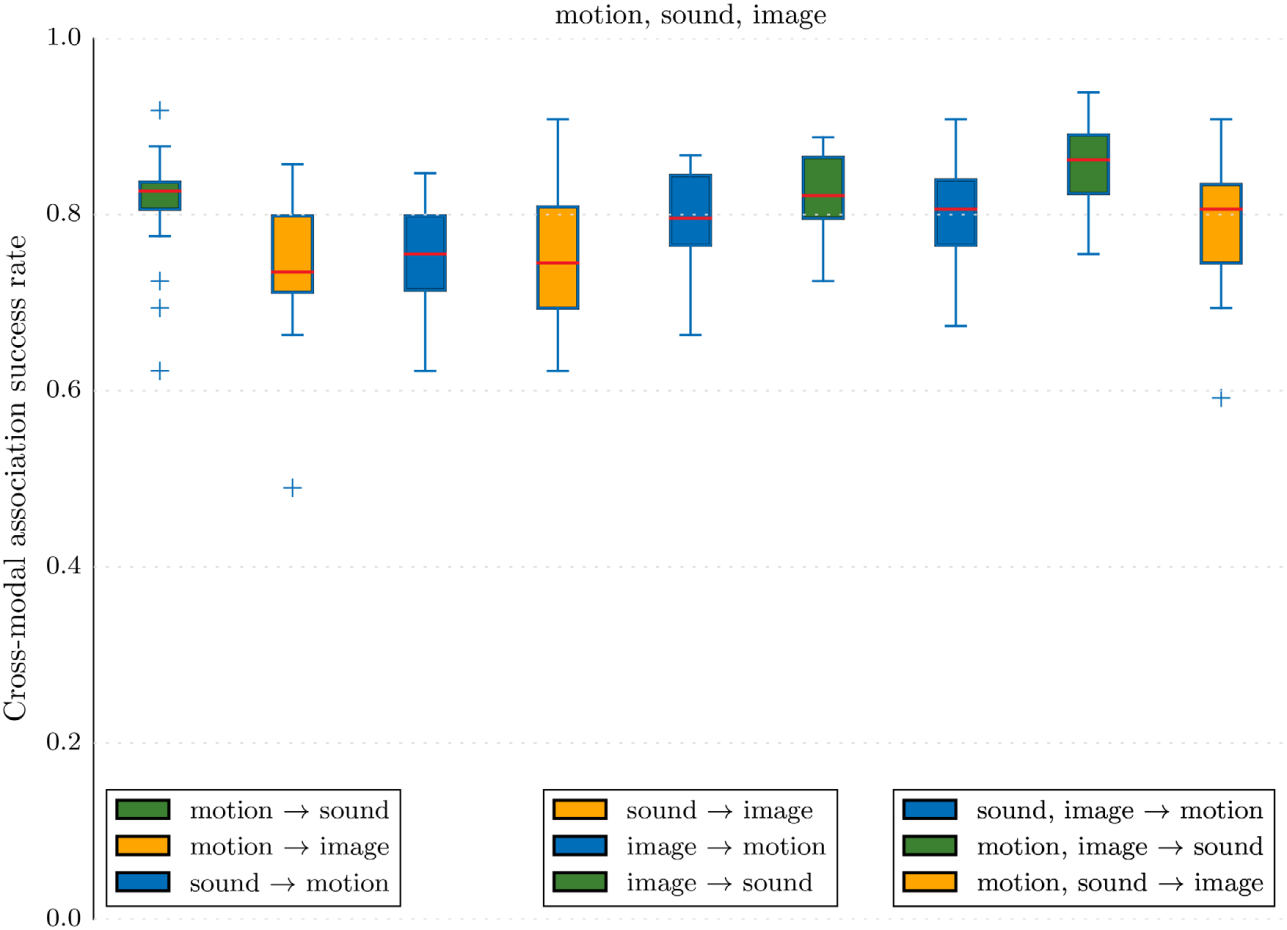
MCA-NMF is capable of relating information from many modalities to one. There is however no substantial improvement in performance from the use of two modalities as input for the recognition. The figure presents box plots of classification success rates for various experiments where three modalities are used for training. There are boxes representing the average success as well as quantiles and extreme values through cross-validation for various testing setups.

Fig 12 presents the results on many possible test setups in the case where all three modalities are present during training. The results demonstrate that the system is capable of using information contained in more than one modality in the test or reference example. Although the results are slightly better when using more modalities as input (as in Motion,Image → Sound in comparison to Motion → Sound or Image → Sound), the improvement in performance is not clearly significant in the experiment.

A last experiment leaves the non-symbolic setup considered previously, in order to compare properties of MCA-NMF with results obtained in previous works, regular classification with the symbolic modality. For example Ngiam et al. [57] present a learner that is trained on multimodal examples of phonemes, either perceived through their acoustic manifestation or through the motions of the lips that pronounce them. In their experiment they show that the learner can benefit from the observation of several modalities and improves its recognition success in comparison to the case where only one modality is observed.

We consider a regular classification setup, similar to the ones presented by Mangin, Driesen et al. [34, 56]. More precisely we introduce a symbolic modality represented by a binary vector as already explained in the previous experiments. The system is trained by observing examples both in the symbolic modality and in one or several other modalities. Then results are compared between various testing setups to explore the ability of the learner to improve its classification performance in the case where several modalities are observed. Such an experiment is actually a classification task with multimodal input unified through *sensor fusion*.

Table 5 presents the results where the sound and motion modalities are combined to a symbolic modality, denoted as L. Interestingly training with the two modalities (sound and motion) does not significantly change the performance of the learner, when tested on sound, motion or both. In that case the benefit of having two non-symbolic modalities is not an increase in performance, but rather that the same learner can use either acoustic perception or motion perception to classify an example.

**Table 5:** Success rate for the label recognition experiment. In this experiment an additional modality containing labels, *L*, is considered. The results are computed on average for a cross-validation of the train and test sets; standard deviations are also given.

Training	Testing	Success rates
S + L	S → L	0.916 ± 0.034
M + L	M → L	0.906 ± 0.052
S + M + L	S → L	0.896 ± 0.043
S + M + L	M → L	0.910 ± 0.054
S + M + L	S + M → L	0.917 ± 0.055

### Word acquisition and recognition

The previous experiments demonstrate that the artificial learner is capable of learning the semantic connection between utterances and the objects or motions they describe. The meaning of the sentences is modelled in our experiment by the presence of a keyword; more precisely the association between sentences and images of objects or motions are based on the presence of keywords in the utterances. However the learner is not aware of the fact that all the meaning of the sentence is actually localized in one word; instead it only exploits cross-situational learning to discover relations between acoustic and, say, visual features. The task solved by the learner actually only involves holistic understanding and classification of the sentences. Therefore it is not completely clear what information the learner actually exploits in the sentence and whether the learner discovers word-like units from the acoustic stream. Indeed the previous experiments only demonstrate that the learner achieves teleological understanding of the sentences; however the question remains to know if it starts to understand compositionally the sentences. We further explore this question in the experiment presented in Section *Words’ location in sentences*, that can be seen as extending the one presented by Stouten et al. [58] to the multimodal setup instead of only speech and labels. The system’s behavior also display that the semantic classes have been encoded in its perception but not how it is encoded. Hence Section *Emergence of localized representations of concepts* takes a further look at this question.

#### Words’ location in sentences

The experiment presented in this section explores whether the meaning of sentences is localized around the keyword that bears this meaning. In this section the system is trained as in the previous experiments but we change the evaluation procedure. The system does not have to relate full utterances to visual objects any more. Instead we extract sliding windows of a given length from the utterances from the test set. The sliding windows extraction process actually takes two parameters: the width of the windows and the shift between two windows. In the following we use sliding windows of width 0.5 s with a 0.05 s shift. Once a sliding window has been extracted, it is represented using the HAC vectors, as previously. Then each sound window is compared by the system to several examples in another modality, here images of objects. By evaluating the system on small parts of utterance we can visualize which part of sentences are associated with each object, that is with each underlying semantic concept.

**Fig 13:**
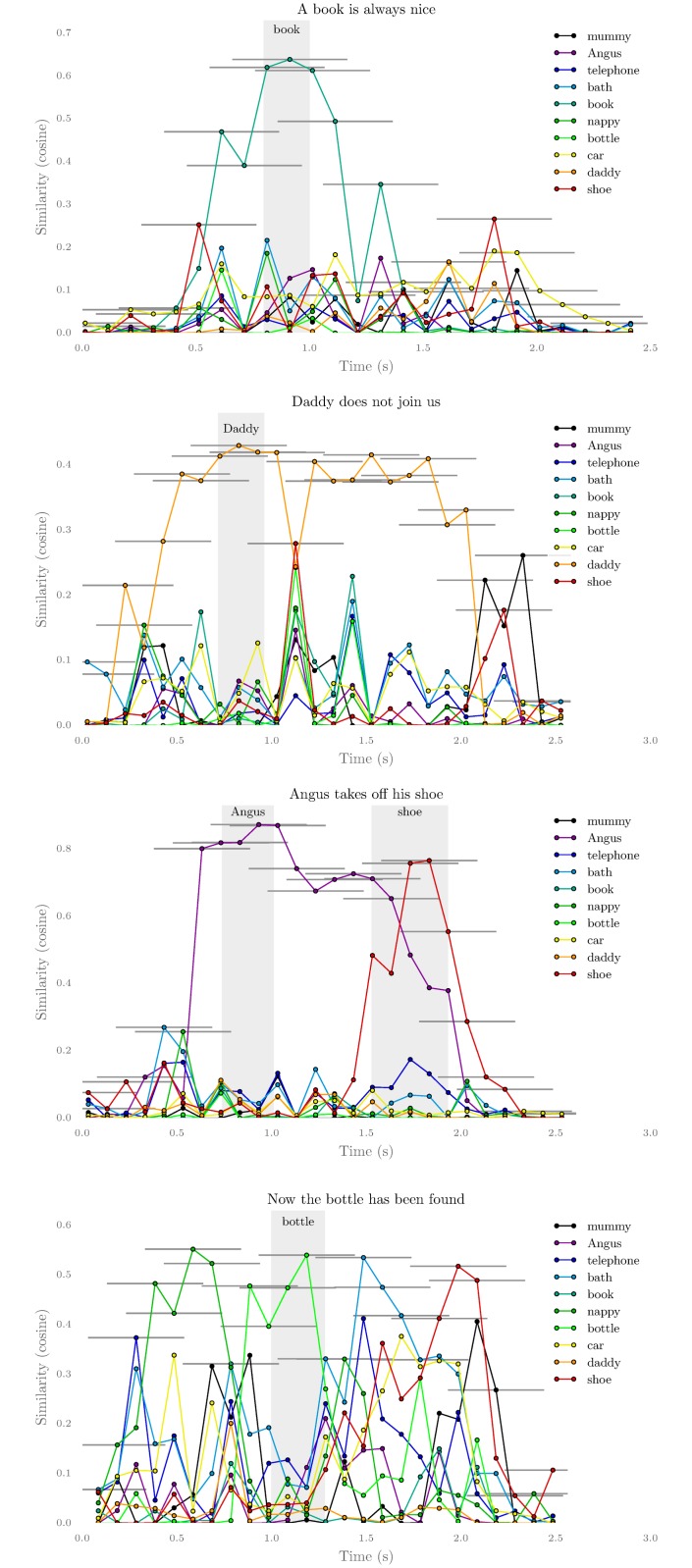
These examples illustrates various distributions of meaning in utterances, in the learner’s point of view. More precisely the horizontal axis represents time: the dots correspond to the mean time of sound windows, which width are materialized by an horizontal grey bar. Thus each plot represents the similarity between the small sound windows and various pictures which semantic class correspond to colors. The four sentences were chosen because they illustrate typical situations. Each record of utterance starts and ends with approximately half a second of silence. Importantly the similarities are taken between sound and images, that is, when a chunk of sound is similar to *‘mummy’*, it actually means that the learner’s associates it strongly with an image of the object representing the *mummy* concept. The location of key words, in grey areas, have been indicated by manually detecting their boundaries in the utterance.

In Fig 13, we present four typical behaviors of MCA-NMF on test sentences. The examples have been chosen to illustrate these typical situations but do not correspond to what four random examples would look like. All these examples are taken from the same learner.

The first example (top left), corresponding to the sentence *‘The book is always nice.’*, features a high similarity between the *book* image and sound windows that intersect the location of the word book in the sentence (between 0.8 and 1.0*s*).

In the second sentence (top right), the correct meaning is guessed by the system but it is not located specifically in the sentence. Because the dataset used for training is rather small in comparison to the variety of word combinations, many non-keywords appears only or most often together with the same keyword. In this case the word *‘join’* and the phrase *‘join us’* are always observed together with *‘daddy’*, while the occurrences of *‘does’* are more spread among the various keywords. The grammar used to generate the utterances is described concisely by Altosaar et al. [36] and in more details by Driesen [50]. This situation is similar to the case of natural sentences where contexts highly modify the distributions of words and may explain such de-localization of the perceived meaning of sentences. In particular this provides an example where the system achieves teleological understanding, which here means guessing the general meaning of a sentence, without compositional understanding.

The third sentence (bottom left) is very specific because it is one of the rare ambiguous sentences of the dataset. Indeed the sentence *‘Angus takes off his shoe.’* features both the keyword *‘Angus’* and the keyword *‘shoe’*. Furthermore the learner is capable in this sentence to recognize both words and localize them roughly at the start and end of the utterance.

Finally, the fourth sentence illustrates the many cases where there are false positives for other meanings or (not in the example) no clear meaning is found by the learner in the sentence.

#### Emergence of localized representations of concepts

In previous experiments we evaluated the learner on concrete tasks that emphasize its ability to relate information from one modality to another. While this demonstrates that the internal representation built by MCA-NMF encodes these concepts, it is however not trivial how it encodes information. In this section we explore the possibility that at least some components of the dictionary matrix are more specialized into some of the semantic classes.

In order to investigate that aspect we quantified the mutual information between the semantic concepts and the coefficients of the internal representation of samples that features the concepts. For each semantic concept *l* and sample *i* we consider the random variables *X*_*l*_ such that $X_{l}^{i}=1$ if and only if the concept *l* appears in sample *i*. For each dimension *j* of the internal representation and each sample *i* we define the random variable $Y_{j}^{i}=h_{j}^{i}$. We then assume that ${\left(X_{l}^{i}\right)}_{i}$ are independent and identically distributed, as well as the ${\left(Y_{j}^{i}\right)}_{i}$. In the following we quantify the dependency between these two variables by looking at the mutual information between them. In information theory, the mutual information *I* is a measure between two random variables *X* and *Y* defined as “the relative entropy [or Kullback-Leibler divergence] between the joint distribution [*p*(*x*, *y*)] and the product distribution *p*(*x*)*p*(*y*)” by Cover and Thomas [59].

$$I\left(X;Y\right)=D_{KL}(p(x,y)∥p(x)p(y))$$

The *X*_*l*_ variables takes binary values but the *Y*_*j*_ are continuous. Therefore we use a quantization of each coefficients of *h* into 10 discrete values in order to approximate the probability distributions *p*(*X*_*l*_), *p*(*Y*_*j*_), and *p*(*X*_*l*_, *Y*_*j*_) by using the samples for 1 ≤ *i* ≤ *N*. Then we compute the mutual information between the discrete, approximated, probability distributions. From this process we obtain a value *I*(*X*_*l*_; *Y*_*j*_) for each pair (*l*, *j*) that quantifies how much information the coefficient *j* captures from the concept *l*.

**Fig 14:**
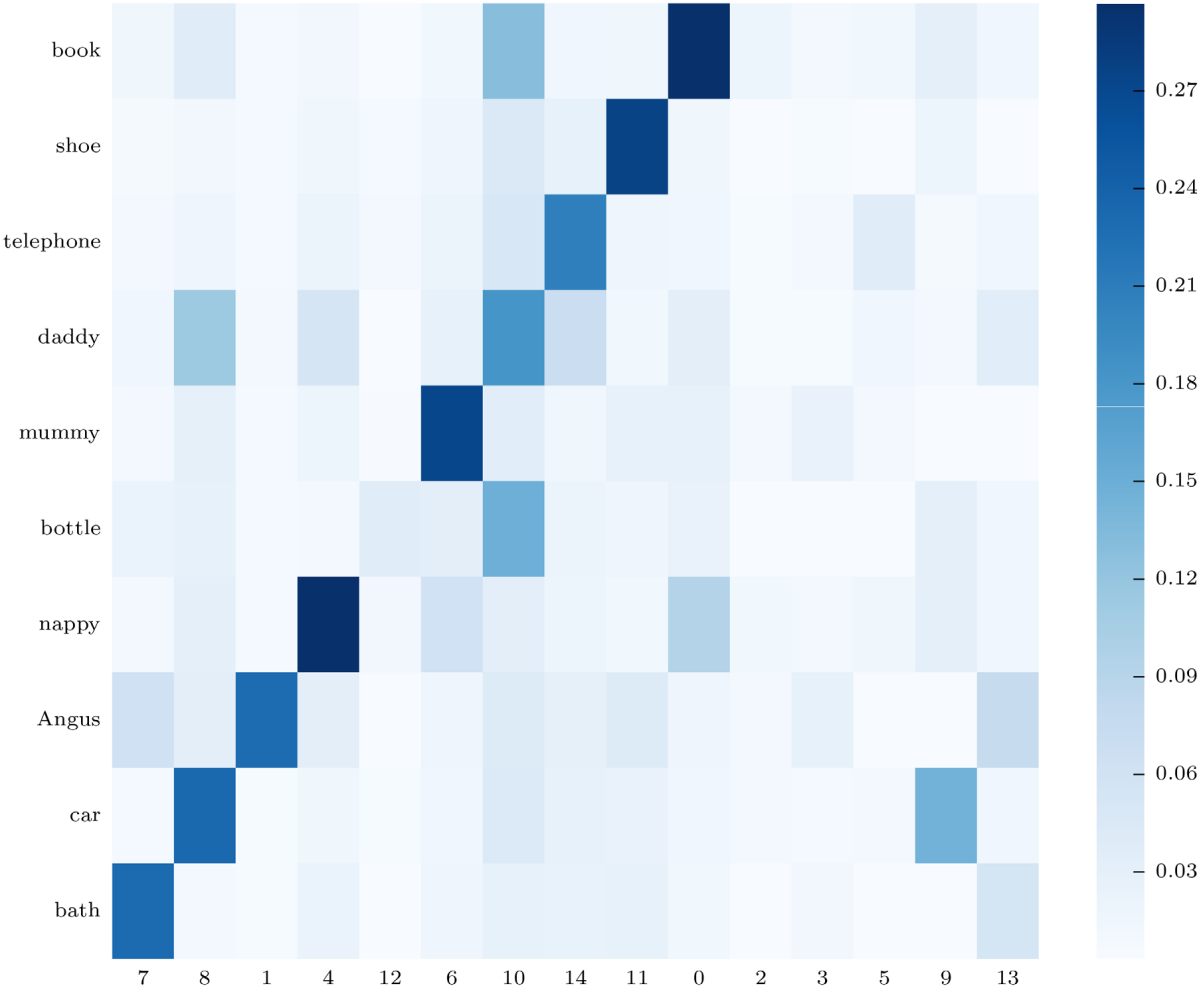
Some components are more associated with some semantic labels. The figure represents the mutual information between (vertically) semantic classes (that are not observed by the learner) and (horizontally) each internal coefficient used by the learner to represent joint occurrences of motion demonstrations, object images, and acoustic descriptions from the training set. A value of *k* = 15 was used in this experiment.

Fig 14 represents the mutual information between each semantic class and each coefficient of the internal representation. To emphasize the specialisation of some internal coefficients we re-ordered internal coefficients so that classes and coefficients that have high mutual information are on the diagonal. More precisely, the best alignment was computed by a Kuhn-Munkres algorithm (see [60]) and we plotted first the coefficients that are highly associated to one class and then the one that are less meaningful. The figure shows that some coefficients are highly specialized in one label. However, it does not display a perfect one to one relationship between labels and coefficients: the information about other labels is spread over several coefficients, and some information is also not clearly localized. For comparison with these results, random labels with uniform probability and a deterministic value of a coefficient knowing the label yields a mutual information of approximately 0.325.

## Discussion and Perspective

In this paper we presented MCA-NMF, a framework for learning multimodal concepts from sensorimotor data, that is based on nonnegative matrix factorization. This algorithm builds a compressed representation of the sensor signals that is exploiting the underlying combinatorial and associative structure of multimodal patterns. The resulting representation is used as an internal representation of the multimodal perception and allows to reconstruct any whole or part of modalities given information on other parts or other modalities.

The experiments presented in this paper demonstrate that this NMF-based procedure is capable of learning concepts from sub-symbolic input streams only. It can overcome several forms of ambiguity, for example related to the discrimination between relevant and non-relevant patterns to encode (such as relevant and non-relevant words in the speech signal). In addition, we discuss the association between patterns from distinct modalities into multimodal concepts.

We then explain how the structure learned from the observation of full, unsegmented, unlabeled sentences can be used to localize *a posteriori* the semantics of the concepts into stretches within the input utterances (such as words). Finally, the last experiment explores how the system internally represents semantic classes. It shows that constraining the representation to be highly compressed leads to a relative specialisation of encoding units into semantic concepts.

### Relation to previous work

Several previous works have addressed related questions to the ones discussed here. As detailed below, the main novelty of our work consists in the facts that 1. we do not rely on symbols for the learning or evaluation phases, 2. the utterances are not segmented into words or phonemes using *a priori* or expert knowledge, 3. more generally, the acoustic modality that contains language is treated symmetrically to the others modalities, 4. the concepts co-organize across modalities, in particular words and their semantics shape each others.

In their seminal work, Roy and Pentland [61, 62] introduce a learning architecture called *Cross-channel early lexical learning* (CELL), together with an example implementation, that demonstrates how the problems of learning *linguistic units*, *semantic categories*, and their relations (in the form of *lexical units*) can be achieved at the same time. CELL involves three stages. It first segments both linguistic information and contextual information, each of which may come from several sensory channels, according to saliency cues such as utterance boundaries or changes in motions. In a second stage, a model of short term memory filters pairs of recurrent co-occurring linguistic and contextual events. Finally a clustering algorithm builds models of linguistic units and semantic categories; they combine clustering of similar language stimuli as well as contextual stimuli and optimize the mutual information between language and context. The pairs of linguistic units and semantic categories with the highest mutual information are kept as lexical units, that correspond to concepts. Thus, in comparison to MCA-NMF, the CELL system first segments sentences in word-like units and thus words and semantics concepts are first formed independently and then filtered and matched, without modelling co-organization of words and their meanings.

Yu and Ballard [63] have presented work addressing a similar problem but focusing more precisely on user-centric and multimodal information. They presented a learning architecture that is capable of forming semantic models of both actions and observed objects by using unsupervised learning techniques. First, models of actions are formed by fitting a mixture of hidden Markov models on the observations and models of objects result from an agglomerative clustering algorithm. The models of objects and actions define concepts and together form the *contextual information*. Then, this contextual information is used to extract word-like units related to these concepts from phoneme transcriptions of the recorded utterances. More precisely, longest common phonetic sequences are extracted from all utterances related to the same object or action. Then an alignment techniques, developed in the field of automatic translation, is used to form the lexical units composed of words and concepts. This approach differs from MCA-NMF since concepts are formed beforehand and the model thus do not take into account the shaping of semantics by the linguistic modality. Furthermore their model relies on mechanisms specific for the linguistic modality as well as expert knowledge to extract phonetics transcriptions.

In Iwahashi [64] the studied language is related to (object, action, position)-based semantics which appears to be closely related to the language grammar. More precisely a lexicon is built from data: the lexicon actually represents a mixture of word and meaning pairs, where meanings can either be objects or actions. Specific probability models are implemented to represent the acoustic modality as well as the modality of visual objects and the one of visual actions. The number of elements in the lexicon is automatically chosen in order to maximize the mutual information between the speech and contextual modalities. Because the acoustic pattern and object recognition abilities are separately acquired in a preliminary stage, this model does not take into account mutual shaping of linguistic and semantics patterns as MCA-NMF. It however features the acquisition of a grammar of the language, learned by identifying in which order the linguistic elements corresponding to the eventual object, action, and landmark appear.

Sugita and Tani [65] introduce a recurrent neural network architecture that learns to relate a basic language to corresponding behaviors of a robot. The system is capable of both understanding the words composing the language, that in their experiment are represented by symbols, and their composition, and so acquires the syntactic structure of the language. As already mentioned in introduction, another aspect of learning action related to language is explored by Tuci et al. [29] who demonstrate that learning a compositional structure shared between action and language can allow better generalization of the motor knowledge. Furthermore Massera et al. [21] have demonstrated that providing linguistic instructions can facilitate the acquisition of a behavioral skill, in comparison to pure motor learning. Although these experiments are limited to symbolic language, they are good illustrations of the implication of learning multimodal actions and grammars.

In our previous work (see [34]), we use the MCA-NMF framework in a multi-label classification experiment. Instead of observing several continuous modalities, the learner observes human demonstrations of motions and a symbolic linguistic modality. However, each demonstration mixes several motions together, forming a complex choreography. Each motion demonstration is thus described in the linguistic modality by several symbols. In other words, there is a joint grammar between the motion and the symbolic modalities. In that experiment, the MCA-NMF system learns the relation between the symbols and the gestures. This relation is initially ambiguous since both gestures and symbols are always observed mixed together with other gestures and symbols, respectively. The experiment then demonstrates that the learner is able to provide correct linguistic descriptions of combinations of gestures that were not observed during training. This result is thus an example of joint grammar acquisition in two modalities, similar to the ones from Sugita, Tuci, Massera et al. [21, 29, 65].

Ten Bosch, Driesen et al. [42, 43] have presented a similar experiment where the contextual (*semantic*) modality is the one that is symbolic and the linguistic (*speech input*) one is continuous. Similarly Lienhart, Akata, BenAbdallah, Srivastava et al. [66–69] use the NMF, probabilistic latent semantic association (PLSA), or deep Boltzmann machine algorithms to learn from a continuous and a symbolic modality. Driesen et al. [56] have also used the NMF algorithm to learn from two continuous modalities. Their model is very close to MCA-NMF and directly inspired it; however, they only evaluate it on the reconstruction of a symbolic modality. An interesting aspect of these approaches is that they all use feature learning algorithms, that are some kind of unsupervised algorithms, instead of relying on explicit models of the lexical units and their relations to language and context.

Another example of the use of feature learning techniques is given by Ngiam et al. [57] who also present an experiment based on a similar multimodal setup. They introduce an architecture based on sparse restricted Boltzmann machines that learns from two continuous modalities: one is acoustic and the other corresponds to the observation of the speaker’s lips. They demonstrate how in certain conditions the algorithm reproduces the McGurk effect. Their algorithm actually learns a new representation of the input in an unsupervised setup and is then evaluated combined with a standard supervised classifier trained on top of this representation. Their work can also be described in terms of sensor-level multimodal fusion: several modalities are used to build a common representation that is later used to solve a classification problem. Multimodal fusion has proven to be a useful technique for improving supervised classification: Potamianos et al. [70] discuss the use of both sensor-level fusion and decision-level fusion for speech recognition. Saenko and Darell [71] also implement decision-level fusion and demonstrate that it improves the recognition of objects. Although closely related to the one of Driesen et al. [56] and similar to the one described in Ngiam et al. [57], the setting of the experiments that we present differs. Indeed, we do not evaluate the learning through a standard classification task: instead of testing the reconstruction of symbolic labels, the system is tested on a behavioral task. In particular this task similar to the ones used to validate children’s learning of concepts, as in Markman, Yurobsky et al. [13, 32].

We show with MCA-NMF that it is not necessary to fit an explicit representation of a lexicon in order to produce behaviors that are considered on children as evidence of children’s acquisition of lexical items. That aspect is an important novelty in comparison to the aforementioned previous work.

The interactions mechanism between the learning agent implemented by MCA-NMF and its caregiver, who provides the demonstrations of sentences, actually shares many similarities with the one from the *Talking Heads* experiment as described by Steels and Kaplan [72, 73]. More precisely the agent we present in this paper plays the role of the *hearer* from the talking heads, while the caregiver takes the role of the *speaker*. There are however important differences between our setup and the one from Steels [72]. First, there is no turn in the role taken in our experiment: the learner only plays the hearer and the caregiver only plays the speaker. Importantly this means that the language is taught to the learner by the caregiver, instead of evolving and emerging from their interaction. Second, in our experiments, the naming game, that consists for the hearer in guessing which object the speaker is talking about, is only played during the evaluation stage. During the training the learner passively observes the caregiver teaching and does not receive any other feedback. These changes make MCA-NMF an alternate implementation of a *naming game* hearer, that does not rely on the success of the naming to update its knowledge, but rather uses cross-modal information to do so. This means here that the cross-modal heuristics may be an alternative to feedback on the game success.

### Interpretations and limitations

In this paper we demonstrate, with MCA-NMF, the possibility of multimodal learning from a flow of non-symbolic sensory input streams by using variations of the non-negative matrix factorization. The ideas we present are however not restricted to this specific algorithm and its implementation. For example, algorithms based on deep belief networks have been shown to perform well on problems similar to the one addressed here, though only on symbolic language, by Srivastava and Salakhutdinov [69]. It is thus an interesting subject for future work to investigate the behavior of these algorithms or other feature learning algorithms on setups similar to this one, as started by Droniou et al. [74]. Also, experiments on other modalities, in particular aiming at investigating the behavior of MCA-NMF or other algorithms in learning situations with more than three modalities are of great interest for future work.

In this paper, we would like to propose a focus shift in the notion of perceptual decomposition. Concerning the acquisition of meaning of words and the structure of utterances, we do not require a preliminary segmentation of utterances into words, nor do we need to assume a lexicon, nor any prespecified pronunication details, in order to learn the meaning of words. Similarly, in our previous work on learning composite choreographies (see [34]), the MCA-NMF framework does not target the representation of individual gestures. Instead, it builds a representation of full body motions and simultaneously learns its correspondence to a linguistic modality. This approach achieves the recognition of composed gestures thanks to a key property: the internal representation, the mapping between the internal representation and the modalities are compatible with the target structure. Furthermore the strong structure in the symbols of the linguistic modality used in Mangin and Oudeyer [34] that describes the motions is sufficient to shape the learned representation of motions so that it encodes the same structure. Here we demonstrate similarly that the structure in a non-symbolic modality containing images of objects is sufficient to shape the perception of speech in order to recognize individual words that correspond to the objects. Thus, in both the speech and motion cases the explicit representation of words or gestures combinations is not set as a feature of the algorithm. It is instead induced by the data.

The absence of any reliance on segmented and labeled input (for example in the form of handcrafted symbol sequences) is also largely in line with the position put forward by Wrede et al. [30]: accuracy of sentence, motion, or scene decomposition is often a complicated objective to target at first. In contrast, simpler goals, such as understanding the broad meaning of a sentence, can first be achieved that later enable to improve on the decomposition capabilities. Results from Section *Words’ location in sentences* are actually in line with this perspective. This is also coherent with the fact that the apparent obviousness of decomposition in perception is often illusory as revealed by many perceptual experiments (see for example [14–16]).

In terms of learning the grammars of motion and language, a top down approach would consist in a strong a priori on the structure to be learned that the algorithms must encode. The question would be: “What is the best algorithm to learn or encode the grammar from the data?” Instead we propose to explore another question: “Which learning mechanism can lead to the emergence of which grammatical structure?” In MCA-NMF, the operations of word or image juxtaposition or motion combination are translated into nonnegative linear combinations of words or images representations. The results from Mangin and Oudeyer [34] demonstrate that this property enables MCA-NMF to learn the grammar of simultaneous motion. However, this form of grammar is obviously limited compared to other ways to compose motions in sequences or subordinated one to another. Thus future work includes the investigation of the ability of this mechanism to represent and let emerge other types of motion or word grammars. From this investigation follows the question of the data that can lead to the learning of new grammars. In particular which grammatical structure are common to several modalities such as speech, vision, or motion perception. Similar future work must also investigate the same question with other combination mechanisms and learning algorithms.

In this paper we show that MCA-NMF is able to acquire word knowledge from cross-situational cross-modal information. The experiments we presented are however limited to data that has been artificially forged to encode cross-situational information. It is thus an essential extension to attempt these learning schemes on realistic data. This constitutes a way to assert the strength of cross-situational information in infant-like perception, and hence to investigate its role in learning infants. Another important extension is the design of experiments on realistic data that use competitive computational models to better delimit what cross-situational information can and cannot explain in word learning. Indeed, many classes of words and meanings may be impossible to learn solely from that information. Finally, the exploration of other word learning heuristics is also a major subject for future work. Indeed, other rules such as mutual exclusivity or whole object assumption have been identified to play an important role in the process of learning words Markman [13]. Their study raises new challenges such as building computational models of learners sensible to such information and experiment their capabilities.

The MCA-NMF model we present in this paper also suffers from important limitations. First, in its current state it does not consider the developmental dimensions of concept acquisition. For example it is based on a batch learning algorithm; although incremental versions can be implemented which yield the same results(see for example [43]), the present paper does not cover this aspect. In particular, we illustrated various behaviors of the learners in the experiments with sliding windows and interpreted them as teleological understanding potentially enabling compositional understanding. Further analysis of the occurrences of such behaviors may benefit from being performed in a dynamical setting, in order to focus on the evolution of the learner through its development. There is no limitation in principle to implement the MCA-NMF learner incrementally, which would enable new experimental possibilities.

Second, we fix the number of atom (internal representations) *k*, whereas the algorithm could adapt it based on the error rate on encoding perception. Another question unexplored in these experiments is the impact of noise in the cross-modal associations on the learning success (see for example [75]). As an example it would enable to study the epigenetic evolution and refinement of a concept.

Third, in our approach we do not address the question of attention. More precisely we implicitly consider two basic models of attention: full utterances and sliding windows. Such restriction on time scales may prevent to account for several aspects of human speech perception and further investigation in this direction also opens perspectives for future work.

Finally, the sensorimotor aspect of perception and other aspects of the interplay between action and perception are out of the scope of this paper but of great interest (see [25]). In particular active attentional mechanisms, and more generally active learning and exploration, play an important role in perception and learning, at different time scales, to shape actively the data perceived by the learner (see [76]). While such aspects were not explored in this paper the proposed MCA-NMF framework and its implementation may further be used as a building block to study these questions.
